# Replacement of Anterior Composite Resin Restorations Using Conservative Ceramics for Occlusal and Periodontal Rehabilitation: An 18-Month Clinical Follow-Up

**DOI:** 10.1155/2016/9728593

**Published:** 2016-07-31

**Authors:** Leonardo Fernandes da Cunha, Rayane Alexandra Prochnow, Adriana Osten Costacurta, Carla Castiglia Gonzaga, Gisele Maria Correr

**Affiliations:** Graduate Program in Dentistry, Universidade Positivo, 5300 Rua Professor Pedro Viriato Parigot de Souza, 81280-330 Curitiba, PR, Brazil

## Abstract

This case report describes a patient with discolored and fractured composite resin restorations on the anterior teeth in whom substitution was indicated. After wax-up and mock-up, the composite was removed and replaced with minimally invasive ceramic laminates. An established and predictable protocol was performed using resin cement. Minimally invasive ceramic restorations are increasingly being used to replace composite restorations. This treatment improves the occlusal and periodontal aspects during the planning and restorative phases, such as anterior guides, and laterality can be restored easily with ceramic laminates. In addition, the surface smoothness and contour of ceramic restorations do not affect the health of the surrounding periodontal tissues. Here we present the outcome after 18 months of clinical follow-up in a patient in whom composite resin restorations in the anterior teeth were replaced with minimally invasive ceramic laminates.

## 1. Introduction

Every patient wants a functional, healthy, and esthetically appealing smile. Nevertheless, restorative cosmetic dentistry should be used conservatively. With improvements in dental ceramics and use of adhesive systems, conservative ceramic laminates are now considered by both dentists and patients to be one of the most viable treatment options in cosmetic dentistry [1].

Minimally invasive ceramic laminates can be indicated when the patient presents with tooth wear or an extensive diastema affecting an anterior composite or the natural teeth. In such cases, little or no preparation of the tooth is necessary. Further, the longevity of adhesion to the enamel has been well established [2].

Failed, discolored, or fractured anterior restorations and damaged teeth have a negative impact on the smile [1]. Therefore, rehabilitation should include reestablishment of occlusion, such as anterior and lateral guidance. Before replacement of a restoration, occlusal guidance can be tested with a mock-up for diagnosis and a temporary restoration [3]. This planning strategy can be used to complement the esthetic intervention and improve occlusal aspects [4].

Gingival health should also be evaluated when esthetic procedures are considered. This is an esthetic consideration that can significantly influence the final result of restoration. Indirect restorations of ceramics, such as veneers, can result in better periodontal health when compared with composite resin and also present an adequate emergence profile [5].

Various materials are available for minimally invasive ceramic restoration. Reinforced ceramic lithium disilicate is commonly used because of its optical and physical properties [6]. The improved mechanical properties of these materials can help dentists and laboratory technicians achieve clinical success.

This case report describes the procedure used to replace a composite resin restoration with minimally invasive laminates in a patient seeking improved occlusal and periodontal smile esthetics, as well as the outcome after 18 months of clinical follow-up.

## 2. Case Report

**Figure 1:**
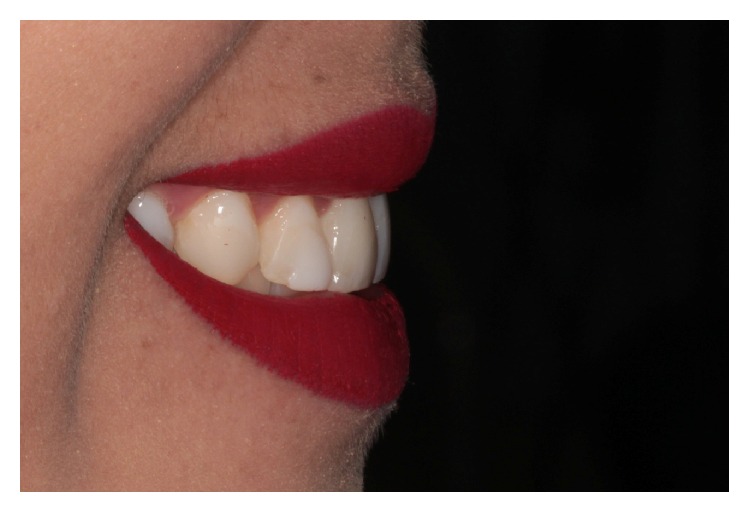
Initial smile of this patient.

**Figure 2:**
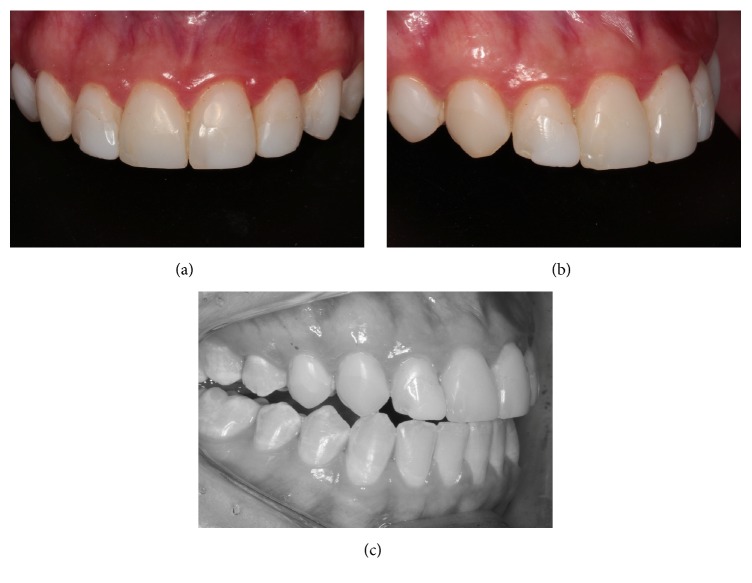
Close-up view of frontal (a) and lateral (b) anterior teeth showing worn and stained composite resins. (c) Image of right disclusion showing contact of canine as well as lateral and central incisors.

A young woman presented at our dental specialties clinic complaining of staining and fractures of the composite resin restorations in her upper anterior teeth. A history and physical examination revealed dental fluorosis and small gaps. Anterior disclusion and laterality were absent (Figures 1 and 2). Gingival inflammation was observed near the borders of the subgingival resin. This was clinically verified based on her symptoms of gingival bleeding and accumulation of plaque.

**Figure 3:**
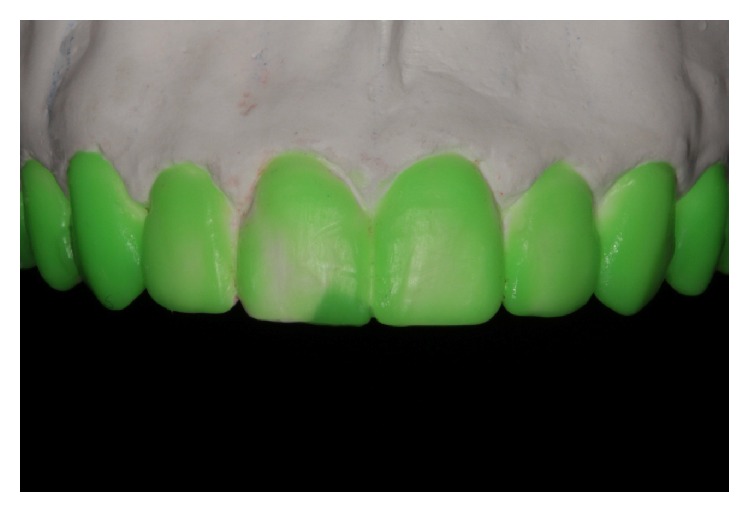
Final diagnostic appearance.

Replacement of the resin restorations by minimally invasive ceramic laminates was proposed to the patient. Initially, orientation of tooth brushing and dental prophylaxis were performed using an ultrasonic instrument. This was followed by scaling and root planning. After 7 days, a wax model was developed for diagnosis (Figure 3). A gypsum model was obtained from a silicon impression (Express*™* XT; 3M ESPE, St Paul, MN, USA), and a wax-up of this model was performed. A silicon impression was then developed from the wax-up.

**Figure 4:**
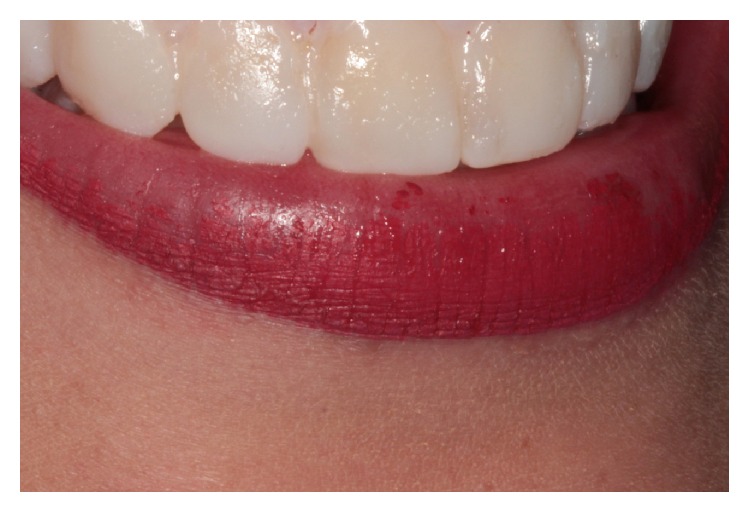
Mock-up made with bis-acryl resin.

In a subsequent session, the color was selected before removal of the composites to prevent dehydration of the substrate. A Protemp 4 (3M ESPE) was inserted into the silicon from the wax-up, and a mock-up was made to assess the size and form of the wax model (Figure 4). This mock-up was also used to evaluate the esthetic length of the tooth and its relationship to the shape of the lower lip and the size of the spaces between the teeth. At that time, the anterior disclusion and laterality guides were evaluated, as well as the emergence profile of the cervical margins of the teeth.

**Figure 5:**
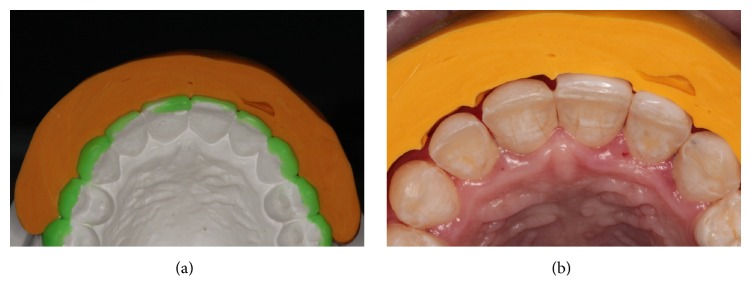
Wear guide made with addition of silicone over wax (a) and on teeth (b) during wear of composite resins to assess thickness of the restoration material.

**Figure 6:**
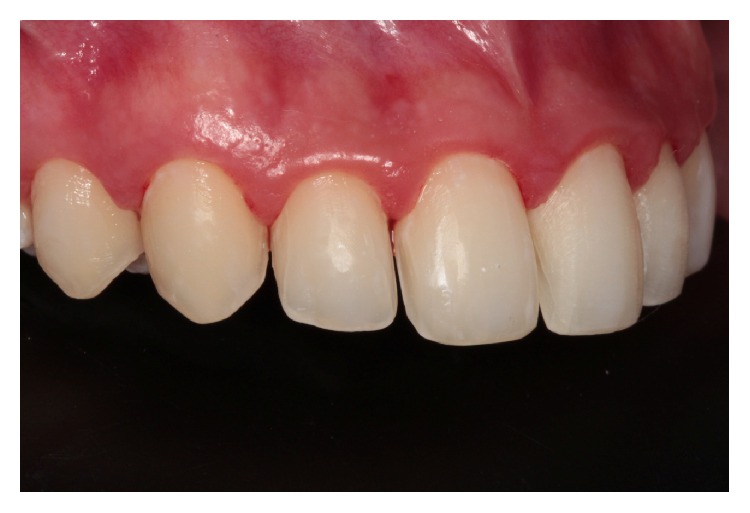
Teeth immediately after preparation. Note gum inflammation caused by the resins.

The restorations were removed using Sof-Lex*™* discs (3M ESPE). After removal of the resin from each tooth, the vestibular space needed for the ceramic was estimated using a wax wear guide (Figure 5). A small cervical demarcation was made in the tooth to establish the completion of indirect restoration. To complete the preparation, the teeth were finished and polished with diamond points using a speed multiplier (W & H Dentalwerk, Bürmoos, Austria; Figure 6).

A Pro-Retract 0000 wire retractor (FGM, Joinville, Brazil) was inserted and a silicone addition impression (Express XT) was performed. Provisional restorations were made using bis-acryl resin (Protemp 4). Provisional restorations made from bis-acryl resin do not need to be cemented. However, the excess gingiva must be removed.

**Figure 7:**
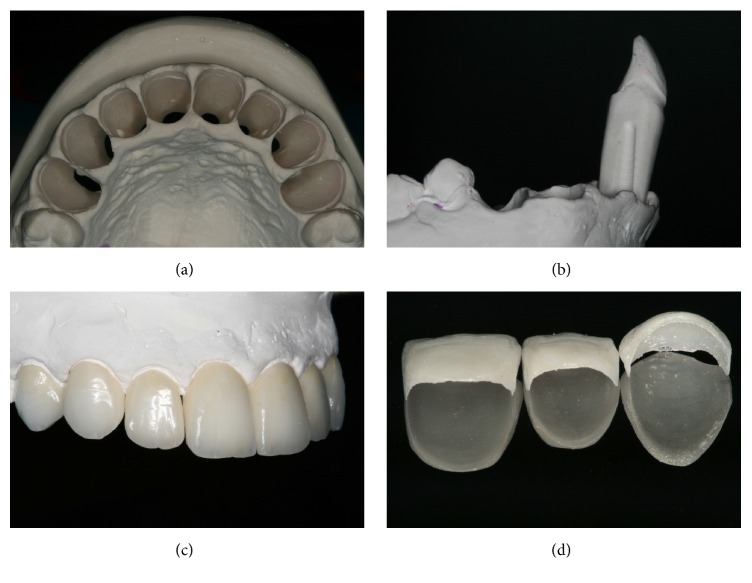
(a)–(d) Model with die in position for creation and ceramic finish to allow for an extremely precise and conservative restoration.

Dies were carefully made to allow for correct construction of the emergence profile. An IPS e.max press (Ivoclar Vivadent, Tamboré, Brazil) was used to make the ceramic laminates (Figure 7). The cervical and proximal finish was performed using stones and ceramic rubber (Zzag, Curitiba, Brazil).

**Figure 8:**
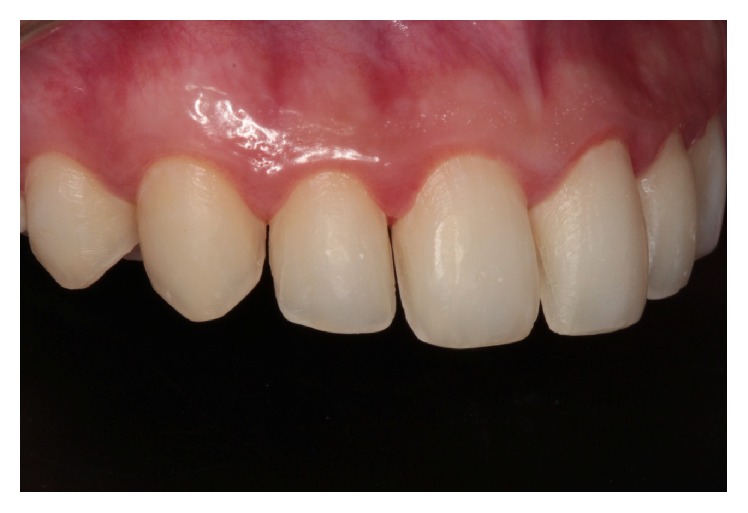
Teeth after removal of temporaries. Note the improvement in papillae after removal of the resins that were causing inflammation.

After a week, the ceramics were tried in the mouth using a try-in clear paste (Nexus*™* 3, Kerr Dental Corporation, Orange, CA, USA). At this time, we could already see an improvement in the appearance of the gingiva after removal of the resins that were causing inflammation (Figure 8). After obtaining approval from the patient, the restorations were etched with hydrofluoric acid for 20 seconds (Condac Porcelain 10%, FGM). Each inner surface was washed for 20 seconds and dried using a triple syringe. Silane (Kerr Dental) was applied as the bonding agent, and 60 seconds were allowed for drying. Adhesive (OptiBond S, Kerr Dental) was applied and polymerization was activated.

We then performed modified tooth isolation, with prophylaxis and etching using phosphoric acid for 30 seconds; the surface was then washed and dried. OptiBond S adhesive was also applied to the teeth and polymerization was activated. Clear cement (Nexus 3) was applied to the inner surfaces of the restorations and placed into position. The excess cement was removed using a microbrush and flossing. Each restoration was cured for 120 seconds using a Radii-cal curing light (SDI, Sao Paulo, Brazil). All materials were applied according to the manufacturer's instructions.

**Figure 9:**
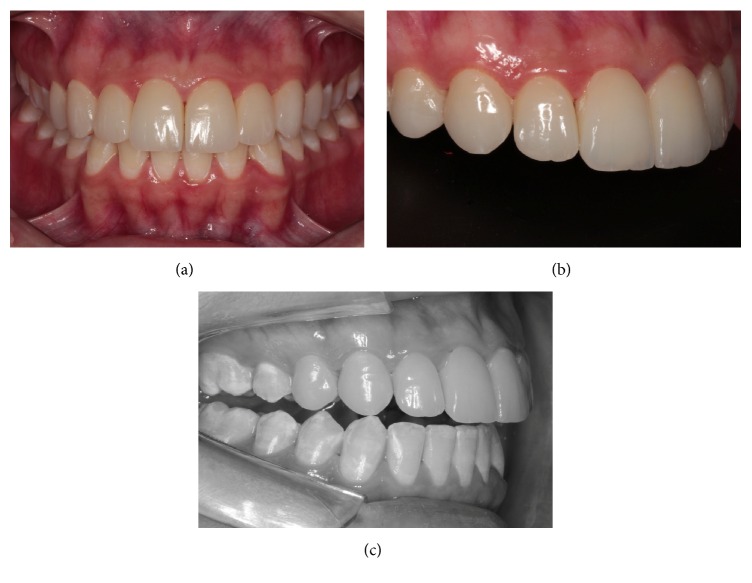
(a)–(c) Ceramic laminates after cementation. Note the disclusion due to the reestablished canine.

**Figure 10:**
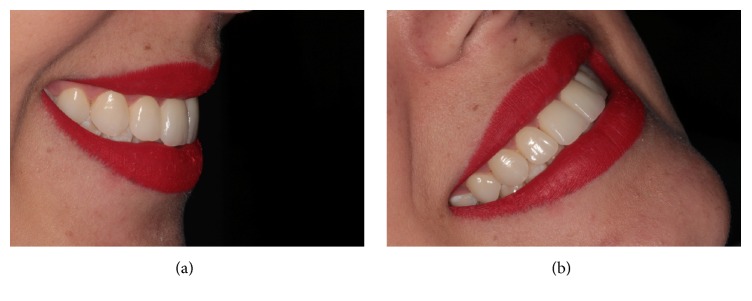
(a)-(b) The final smile achieved for this patient.

**Figure 11:**
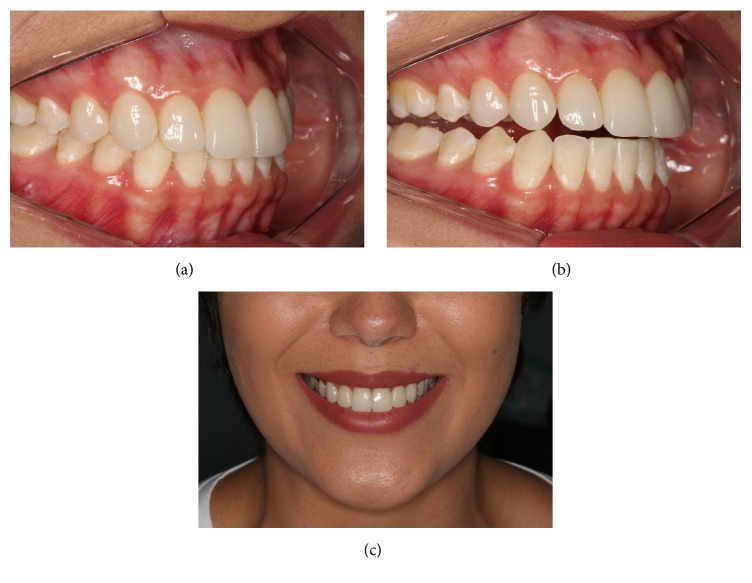
(a), (b), (c) Occlusal and periodontal control and esthetic stability of restorations after 18 months of follow-up.

An occlusal adjustment with ceramic rubber was prepared and checked against the anterior disclusion and laterality guides (Figure 9). The final smile in this patient is shown in Figure 10 and the outcome at 18 months can be seen in Figure 11. After 18 months, there was no clinical evidence of gingival bleeding or accumulation of plaque.

## 3. Discussion

A number of restorative approaches could have been used in this patient, including direct composite resins and minimally invasive ceramic laminates. The success of resin depends on the skill of the operator and the esthetic wishes of the patient. However, long-term clinical outline, color stability, durability, and occlusion are critical for the anterior teeth. Currently, porcelain veneers afford predictable and successful restoration, with an estimated approximate survival of 10 years [7]. In the present case, there was a good amount of sound structure and no significant color change, so ceramic laminates were a suitable option. After being informed about the advantages and disadvantages of each type of restoration, the patient opted for conservative ceramic veneers of minimum thickness.

A pressed glass-ceramic laminate was used in this patient. For esthetic veneers, ceramics reinforced by leucite, such as VITAPM® 9 (VITA Zahnfabrik, Bad Säckingen, Germany), and those reinforced by lithium disilicate, such as IPS e.max, are good examples and are commonly indicated because of their optical and mechanical [6] properties. Leucite and lithium disilicate particles are added to the base glass composition of these ceramics to improve their resistance to fracture without impairing their optical properties. It is also important to note that the mechanical properties of these materials depend on the shape and volume of the crystals, among other factors [8, 9]. These properties provide sufficient strength to withstand anterior and lateral disclusion when compared with direct composite resins such as those that had initially been used in this patient. Due to the relatively low refractive index of leucite and lithium disilicate, even with a relatively high crystalline content, these materials may be considered translucent and esthetic [1]. Consequently, optical effects, such as opalescence, color, and opacity, are excellent for restoring translucency to the incisal edge, as seen in our patient. Finally, these materials are biocompatible restorative materials that improve periodontal health in the long term due to their surface smoothness.

Esthetic treatments should not be performed without appropriate restoration planning. Gurel et al. [4] demonstrated the use of mock-up and temporary techniques for preoperative evaluation and as a diagnostic aid for the final result, as in the present case, where the mock-up was used to facilitate communication with the patient in the diagnostic phase. It was also used to demonstrate the esthetic design of the new emergence profile to improve the incisal reestablishment with the lips and the anterior and lateral disclusion guides. Consequently, the guide used for mock-up can also be used for the manufacture of a temporary restoration.

Biomechanical and occlusal principles that can help optimize the conservative treatment of worn teeth should be selected. According to Abduo et al. [10], during full excursion, canine-guided occlusion tends to be more frequently observed; with aging, the prevalence of canine-guided occlusion tends to be reduced, and that of group function occlusion increases. In this case, the patient was very young and the composite resin in her anterior teeth had almost certainly worn down. Therefore, esthetic restoration was performed with ceramics. Moreover, ceramic materials perform better in terms of discoloration, integrity of the margins, minor fractures, and cracking when compared with composite resin.

Gingival health should always be evaluated. Periodontal health before restorative treatment is important [11], and pretreatment with prophylaxis and scaling will improve the impressions taken of the teeth. Well-adapted temporaries without excess material will facilitate the cementing process, thus improving periodontal health in the long term [5]. This can be considered to be an ideal periodontal condition to prepare the teeth and obtain impressions in the absence of gingival bleeding and accumulation of plaque. This will also improve the prognosis of the treatment.

The preparation was restricted to the enamel and was therefore conservative, favoring adhesive cementation [12, 13]. Retention of the restoration is also helped by use of hydrofluoric acid to condition the inside surfaces and by use of silane coupling agents. A photopolymerizable resin cement should be used for thin restorations, because these are translucent [1, 10]. This also shortens the treatment time. In addition, for esthetic reasons, this system includes a try-in paste, so the final result is more predictable [1].

The ultimate success of functional and esthetic treatment depends on the patient being well informed and motivated to maintain oral health. Cooperation on the part of the patient and periodic intervention by the dentist are essential for long-term success of the restoration [1, 6, 7]. After 18 months of clinical follow-up, the restorations in our patient proved to be adequate from both the functional and esthetic points of view, with maintenance of occlusion and periodontal health.

## 4. Conclusion

Replacement of resin composites by minimally invasive ceramic laminates can rehabilitate the teeth in a safe and esthetically pleasing manner. When carried out appropriately, occlusion and periodontal health can also be reestablished.
